# Follicular extracellular vesicles enhance meiotic resumption of domestic cat vitrified oocytes

**DOI:** 10.1038/s41598-020-65497-w

**Published:** 2020-05-25

**Authors:** Marcia de Almeida Monteiro Melo Ferraz, Mayako Fujihara, Jennifer Beth Nagashima, Michael James Noonan, Miho Inoue-Murayama, Nucharin Songsasen

**Affiliations:** 1 Smithsonian National Zoo and Conservation Biology Institute, 1500 Remount Road, Front Royal, Virginia 22630 USA; 2 Wildlife Research Center, Kyoto University, 2-24 Tanaka-Sekiden-cho, Sakyo Kyoto, 606-8203 Japan; 3 Wildlife Genome Collaborative Research Group, National Institute for Environmental Studies, 16-2 Onogawa, Tsukuba Ibaraki, 305-8506 Japan

**Keywords:** Proteomic analysis, Cell delivery

## Abstract

Extracellular vesicles (EVs) contain multiple factors that regulate cell and tissue function. However, understanding of their influence on gametes, including communication with the oocyte, remains limited. In the present study, we characterized the proteome of domestic cat (*Felis catus*) follicular fluid EVs (ffEV). To determine the influence of follicular fluid EVs on gamete cryosurvival and the ability to undergo *in vitro* maturation, cat oocytes were vitrified using the Cryotop method in the presence or absence of ffEV. Vitrified oocytes were thawed with or without ffEVs, assessed for survival, *in vitro* cultured for 26 hours and then evaluated for viability and meiotic status. Cat ffEVs had an average size of 129.3 ± 61.7 nm (mean ± SD) and characteristic doughnut shaped circular vesicles in transmission electron microscopy. Proteomic analyses of the ffEVs identified a total of 674 protein groups out of 1,974 proteins, which were classified as being involved in regulation of oxidative phosphorylation, extracellular matrix formation, oocyte meiosis, cholesterol metabolism, glycolysis/gluconeogenesis, and MAPK, PI3K-AKT, HIPPO and calcium signaling pathways. Furthermore, several chaperone proteins associated with the responses to osmotic and thermal stresses were also identified. There were no differences in the oocyte survival among fresh and vitrified oocyte; however, the addition of ffEVs to vitrification and/or thawing media enhanced the ability of frozen-thawed oocytes to resume meiosis. In summary, this study is the first to characterize protein content of cat ffEVs and their potential roles in sustaining meiotic competence of cryopreserved oocytes.

## Introduction

Assisted reproductive technologies (ARTs), especially the cryopreservation of gametes, are useful tools for preserving the fertility of human patients undergoing chemotherapy treatment^1^. Beyond their importance in human applications, ARTs are also a critical tool for the genetic preservation and *ex situ* population management of endangered species^2,3^. Embryo and sperm cryopreservation technologies are well established and routinely used in human fertility clinics^4^. Unlike sperm and embryos, the oocyte has several unique features (e.g., large size and amount of intracellular lipid) that contribute to its extreme susceptibility to damage during cryopreservation^5,6^. Nevertheless, the development of minimum volume vitrification (MVV) methods, such as open pulled straw and Cryotop, which permit cooling rates exceeding −100,000 °C min^−1^, has significantly improved the survival and function of frozen-thawed gametes^7,8^. So far, live offspring have been produced from cryopreserved mature oocytes in several mammalian species, including humans^9–12^. Crucially, however, the cryopreservation of immature oocytes is still far from being efficient^4,10,13^. Data from mouse and human studies have shown that vitrification better protects oocytes from structural damage and sustains gametes’ developmental competence than slow freezing^12,14^. For the domestic cat, although both slow-freezing and vitrification have been used to preserve immature oocytes^4,13,15,16^, the rates of cryopreserved immature oocytes that complete nuclear maturation are much lower than for fresh gametes (0–38%)^3,4,13,16,17^. Different approaches have been used to improve the survival and developmental competence of mature and immature vitrified oocytes. These include varying cryoprotectants (CPA) concentrations and exposure times^18–21^, polarization of lipid droplets by centrifugation^9,22^, supplementing freezing media with macromolecules^23^, ice-blockers^20^, or cytoskeleton modifiers^18^, modifications of membrane constituents^19,24^, as well as automation of the addition and removal of cryoprotectants using microfluidics devices^24,25^.

Additionally, it has been shown that human oocytes vitrified in autologous follicular fluid (FF) developed embryos after conventional IVF, with subsequent embryo-transfer resulting in the birth of healthy babies^10^. Follicular fluid is a complex biological fluid that is in close proximity to the developing oocyte^26,27^. The major components of FF are nucleic acids, ions, metabolites, steroid hormones, proteins, reactive oxygen species, polysaccharides and antioxidant enzymes, all of which play important roles in regulating folliculogenesis^26,28^. Recently, extracellular vesicles (EVs), which are likely secreted primarily by the follicle’s granulosa and theca cell populations, have also been detected in FF^28–33^. Extracellular vesicles are membrane encapsulated particles containing regulatory molecules, including proteins, peptides, RNA species, lipids, DNA fragments and microRNAs^34–36^. For follicular fluid EVs (ffEVs), microRNA content has been well-characterized^28,32,37–39^. It has been indicated that microRNAs in ffEVs play an important role regulating expression of genes involved in stress response, cumulus expansion and metabolic functions^31^. Yet, little is known about protein content in ffEVs. A study in the mare has identified 73 proteins in ffEVs, with immunoglobulins being the most abundant^32^. To date, there have been numerous reports on the characteristics of EVs recovered from male and female reproductive tract fluids, including fluids from the prostate^40^, epididymis^40,41^, vagina^42,43^, endometrium^44–46^ and oviduct^47–50^, and their roles in physiologic and pathologic processes^29,31,33,42,50–52^. Yet, the role of ffEVs in protecting oocytes against cryoinjuries has not been explored.

Cat oocytes share many characteristics with human ova, including germinal vesicle chromatin configuration, preovulatory oocyte size, and time to meiotic maturation *in vitro*^53,54^. Here, we utilized the domestic cat as a large-mammalian model with application potential to both endangered felid assisted reproduction and human fertility preservation. In the present study, we characterized the protein content of cat ffEVs and assessed their influence on the survival and *in vitro* maturation potential of vitrified immature cat oocytes.

## Results and discussion

### Follicular fluid EVs characterization

**Figure 1:**
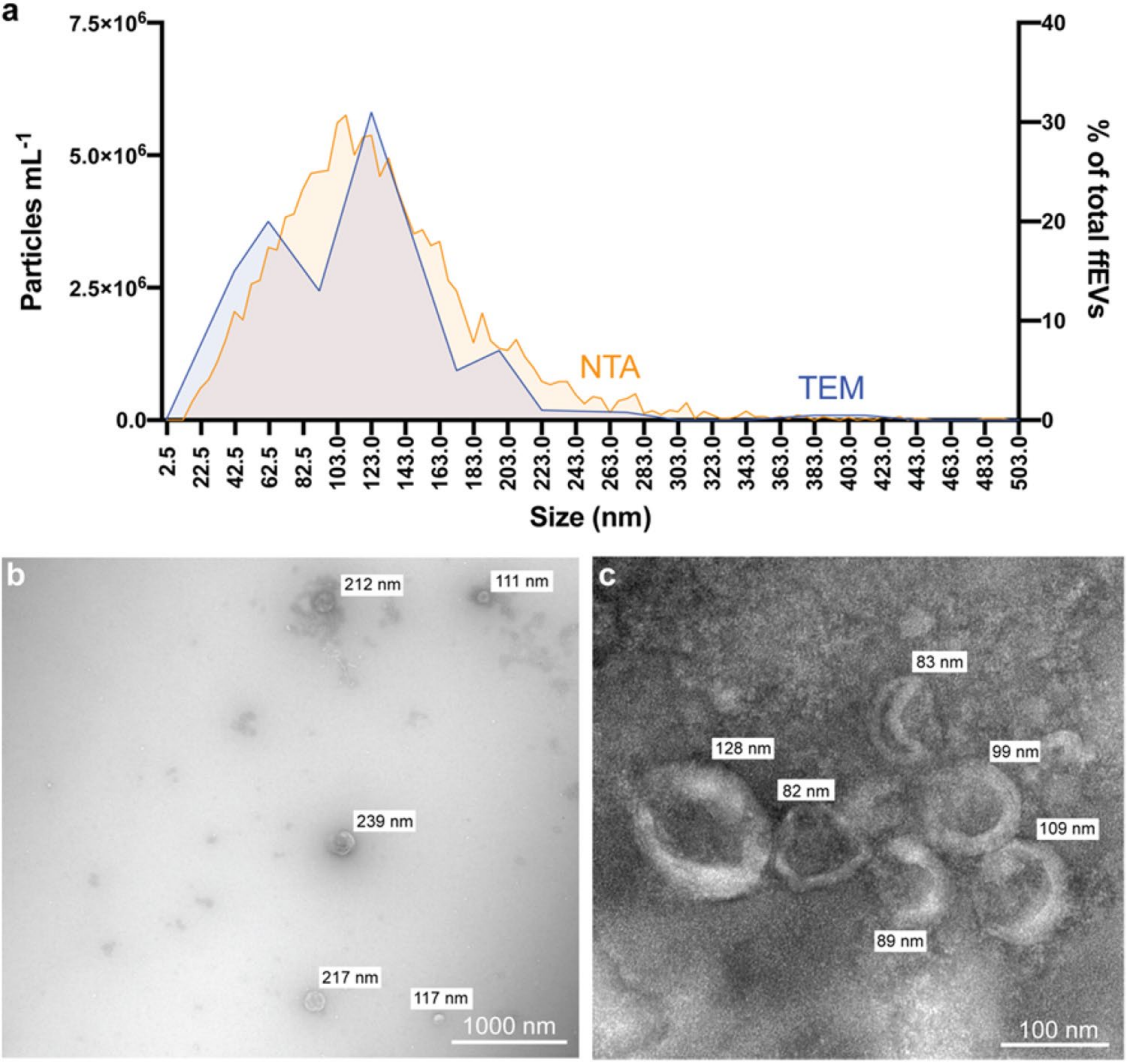
Follicular fluid EVs characterization. In **a** size distribution of ffEVs quantified by nanoparticle tracking analysis (NTA, orange, left y axis), and analyzed by transmission electron microscopy (TEM, in percentage of total counted ffEVs, blue, right y axis). In **b** and **c** TEM image of ffEVs showing different size distribution of isolated ffEVs.

The Total Exosome Isolation Kit (Invitrogen, USA) was used to recover cat ffEVs, as previously utilized for cat oviductal EVs^50^. A combination of Nanoparticle Tracking Analysis (NTA) and Transmission Electron Microscopy (TEM) were used to confirm the presence of, characterize, and quantify cat ffEVs. Zeta View NTA showed the presence of EVs with an average size of 129.3 ± 61.7 nm (Fig. 1a). TEM confirmed the presence of circular vesicles with the characteristic doughnut shape with an average size of 93.2 ± 76.5 nm (12 to 507 nm, Fig. 1a–c). The average size of cat ffEVs observed in the present study is consistent with that reported in the cow (*Bos taurus;* 128–142 nm)^29,33^. The total ffEVs concentration detected by NTA ranged from 1.4 ×10^10^ to 14.0 ×10^10^ particles mL^−1^, with an average of 6.3 ± 5.8 ×10^10^ particles mL^−1^.

In the present study, we used ultraperformance liquid chromatography and tandem mass spectrometry (UPLC-MS/MS) to identify the protein content of follicular fluid EVs. A total of 674 protein groups out of 1,974 protein entries were identified and analyzed for gene ontology (Supplementary Data 1). Two functionally grouped gene ontology (GO) cellular component pathways related to EVs were identified: (1) extracellular vesicle (GO:1903561) and (2) extracellular exosome (GO:0070062); each pathway had 226 and 228 total proteins, respectively. The cat ffEVs contained several EVs markers, including, cytosolic proteins (CHMP4A, PDCD6IP, EHD2, RHOD, ANXA2, ANXA4, ANXA5, ANXA6, ANXA11, HSP90AB1, HSPA8, ACTBL2, ACTG1, ACTR1A, ACTR1B, ACTN2, ACTN1, ACTN4, ACTR3, ACTG1, ACTG2, ACTA1, ACTA2, ACTC1, ACTB, TUBB1, TUBB6, TUBAL3, TUBB4B, TUBB, TUBA8, TUBA4A, TUBB2A, TUBB2B, GAPDH, and GAPDH5), and transmembrane- or lipid-bound extracellular proteins (GNA12, GNA13, GNAT1–3, GNAO1, GNAL, GNAI1–3, ITGB1, ITGA6 and LAMP1), confirming the presence of EV origin in the recovered follicular fluid samples^55^.

Notably, it has been shown that the isolation of extracellular vesicles using precipitation methods, as the one used in the present study, can lead to a higher number of non-EV co-precipitates^55^. Following the Minimal Information for Studies of Extracellular Vesicles guidelines (MISEV 2018)^55^, we identified different kinds of apolipoproteins as potential co-precipitates in the ffEVs. However, apolipoproteins also play a significant role in fertilization and embryo development. It is therefore likely that reproductive fluid EVs naturally contain apolipoproteins, unlike other, non-reproductive EVs used in the MISEV guidelines. In this regard, apolipoproteins have been identified in proteomics of female fluids/EVs or produced by embryos in numerous species, including from EVs of the porcine endometrium^46^, sheep conceptus^56^, sheep uterine fluid of pregnant and non-pregnant sheep^44^, cat oviduct^50^, and from human follicular fluid^57^. In the present context, it is therefore difficult to ascertain if apolipoproteins are co-precipitates or are normally present in reproductive EVs.

### Follicular fluid EVs protein content and their possible role on oocyte structure and function

To further characterize domestic cat ffEV proteins, a functional analysis of the 1,974 protein entries was evaluated using the Cytoscape ClueGO plugin^58^. The comparative analysis of GO terms identified a total of 429 GO biological processes, 136 GO molecular functions, and 151 GO cellular components by analyzing the corresponding genes to all identified proteins in the cat ffEVs in reference to the domestic cat (*Felis catus*) genome (Supplementary data 1). For GO biological processes, 54 terms important for the maintenance of COCs structure and function were identified (Table 1).

**Table 1:** Selected gene ontology (GO) terms for biological processes of ffEVs, including significance (Group P-value) and the percentage of associated genes.

GO ID	GO Term	Group P-value	% Associated Genes
GO:0051276	chromosome organization	0.00	8.29
GO:0051293	establishment of spindle localization	0.00	20.00
GO:0051653	spindle localization	0.00	17.14
GO:0006997	nucleus organization	0.02	8.75
GO:0007051	spindle organization	0.01	8.26
GO:0000212	meiotic spindle organization	0.04	20.00
GO:0051294	establishment of spindle orientation	0.05	12.00
GO:0007010	cytoskeleton organization	0.00	6.69
GO:0030036	actin cytoskeleton organization	0.00	7.42
GO:0007015	actin filament organization	0.00	8.80
GO:0030029	actin filament-based process	0.00	6.84
GO:0031032	actomyosin structure organization	0.00	10.40
GO:0061572	actin filament bundle organization	0.01	8.25
GO:0051017	actin filament bundle assembly	0.01	8.25
GO:0000226	microtubule cytoskeleton organization	0.04	5.31
GO:0031032	actomyosin structure organization	0.00	10.40
GO:0031122	cytoplasmic microtubule organization	0.04	9.76
GO:0030865	cortical cytoskeleton organization	0.02	12.12
GO:0030866	cortical actin cytoskeleton organization	0.02	10.34
GO:0032964	collagen biosynthetic process	0.02	25.00
GO:0032963	collagen metabolic process	0.02	13.51
GO:0032965	regulation of collagen biosynthetic process	0.05	18.18
GO:0010898	positive regulation of triglyceride catabolic process	0.02	40.00
GO:0010873	positive regulation of cholesterol esterification	0.02	33.33
GO:0010896	regulation of triglyceride catabolic process	0.02	28.57
GO:0051006	positive regulation of lipoprotein lipase activity	0.02	28.57
GO:0045723	positive regulation of fatty acid biosynthetic process	0.02	28.57
GO:0034370	triglyceride-rich lipoprotein particle remodeling	0.02	25.00
GO:0034380	high-density lipoprotein particle assembly	0.02	25.00
GO:0034372	very-low-density lipoprotein particle remodeling	0.02	25.00
GO:0010872	regulation of cholesterol esterification	0.02	25.00
GO:0061365	positive regulation of triglyceride lipase activity	0.02	25.00
GO:0033700	phospholipid efflux	0.02	20.00
GO:0034433	steroid esterification	0.02	18.18
GO:0051004	regulation of lipoprotein lipase activity	0.02	18.18
GO:0034434	sterol esterification	0.02	18.18
GO:0034435	cholesterol esterification	0.02	18.18
GO:0043691	reverse cholesterol transport	0.02	18.18
GO:0090208	positive regulation of triglyceride metabolic process	0.02	16.67
GO:0044241	lipid digestion	0.02	14.29
GO:0098609	cell-cell adhesion	0.00	6.30
GO:0034109	homotypic cell-cell adhesion	0.00	21.43
GO:0090136	epithelial cell-cell adhesion	0.01	25.00
GO:0061077	chaperone-mediated protein folding	0.00	21.05
GO:0016485	protein processing	0.00	10.06
GO:0051604	protein maturation	0.00	9.55
GO:0006956	complement activation	0.00	29.41
GO:0072376	protein activation cascade	0.00	40.00
GO:0015671	oxygen transport	0.00	44.44
GO:0045454	cell redox homeostasis	0.00	14.04
GO:1902883	negative regulation of response to oxidative stress	0.01	15.38
GO:1900408	negative regulation of cellular response to oxidative stress	0.01	15.38
GO:0080134	regulation of response to stress	0.00	5.42
GO:0034976	response to endoplasmic reticulum stress	0.00	8.38

**Figure 2:**
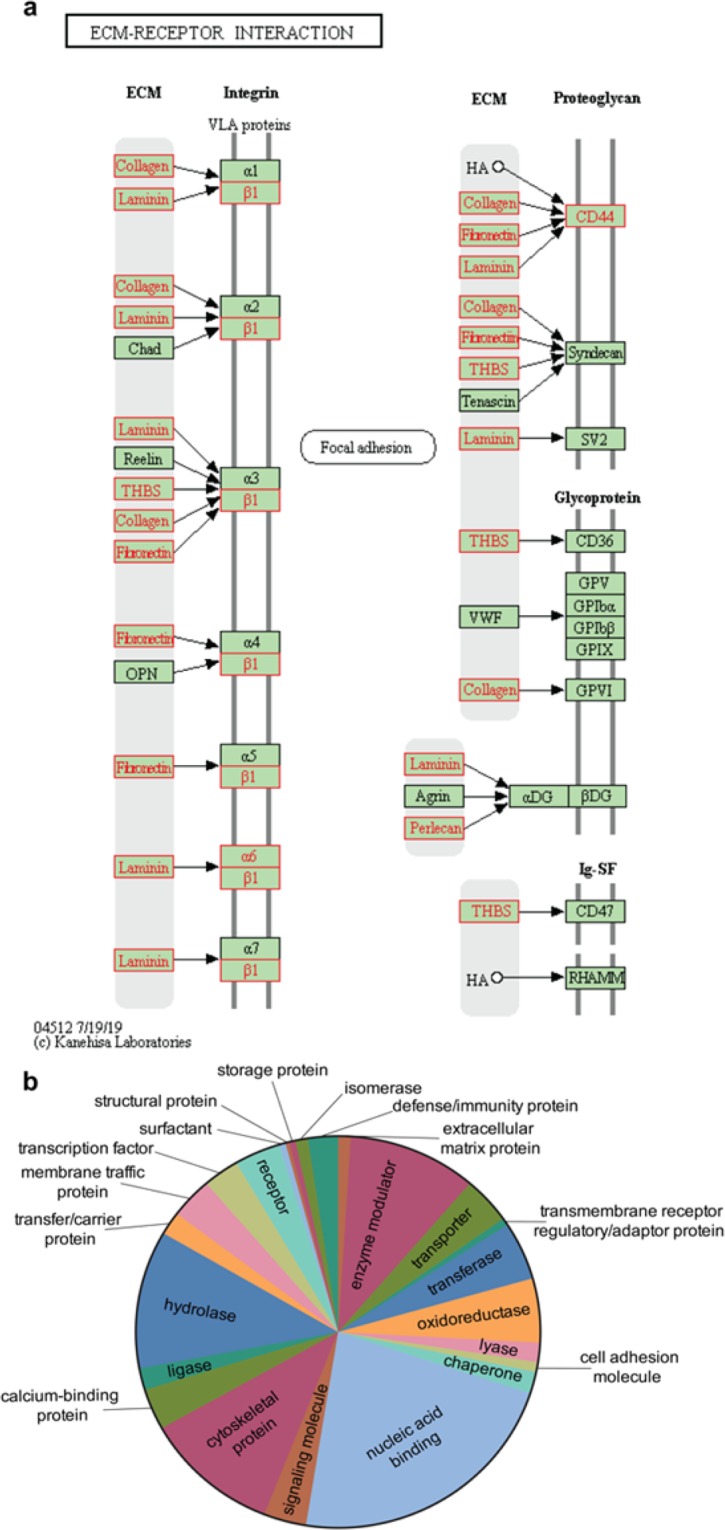
KEGG molecular pathways performed by KEGG mapper (http://www.genome.jp/ kegg/mapper)^59^ and GO protein class of ffEVs identified proteins. In **a,** KEGG pathways related to ECM-receptor interaction. Note that proteins present in ffEVs are shown in red. In **b**, pie chart of the 24 different GO protein classes corresponding to the proteins present in the cat ffEVs.

Next, we identified molecular networks in which ffEVs proteins are active via KEGG mapper^59–62^. Among the identified pathways, oxidative phosphorylation (Supp. Figure 1a), extracellular matrix (ECM, Fig. 2a), oocyte meiosis (Supp. Figure 1b), tight junction (Supp. Figure 2a), regulation of actin cytoskeleton (Supp. Figure 2b), cholesterol metabolism (Supp. Figure 3a), glycolysis/gluconeogenesis (Supp. Figure 3b), and MAPK, PI3K-AKT, HIPPO and calcium signaling pathways (Supp. Figure 4a–d) have been shown previously to play important roles in oocyte structure and function^63–69^.

**Figure 3:**
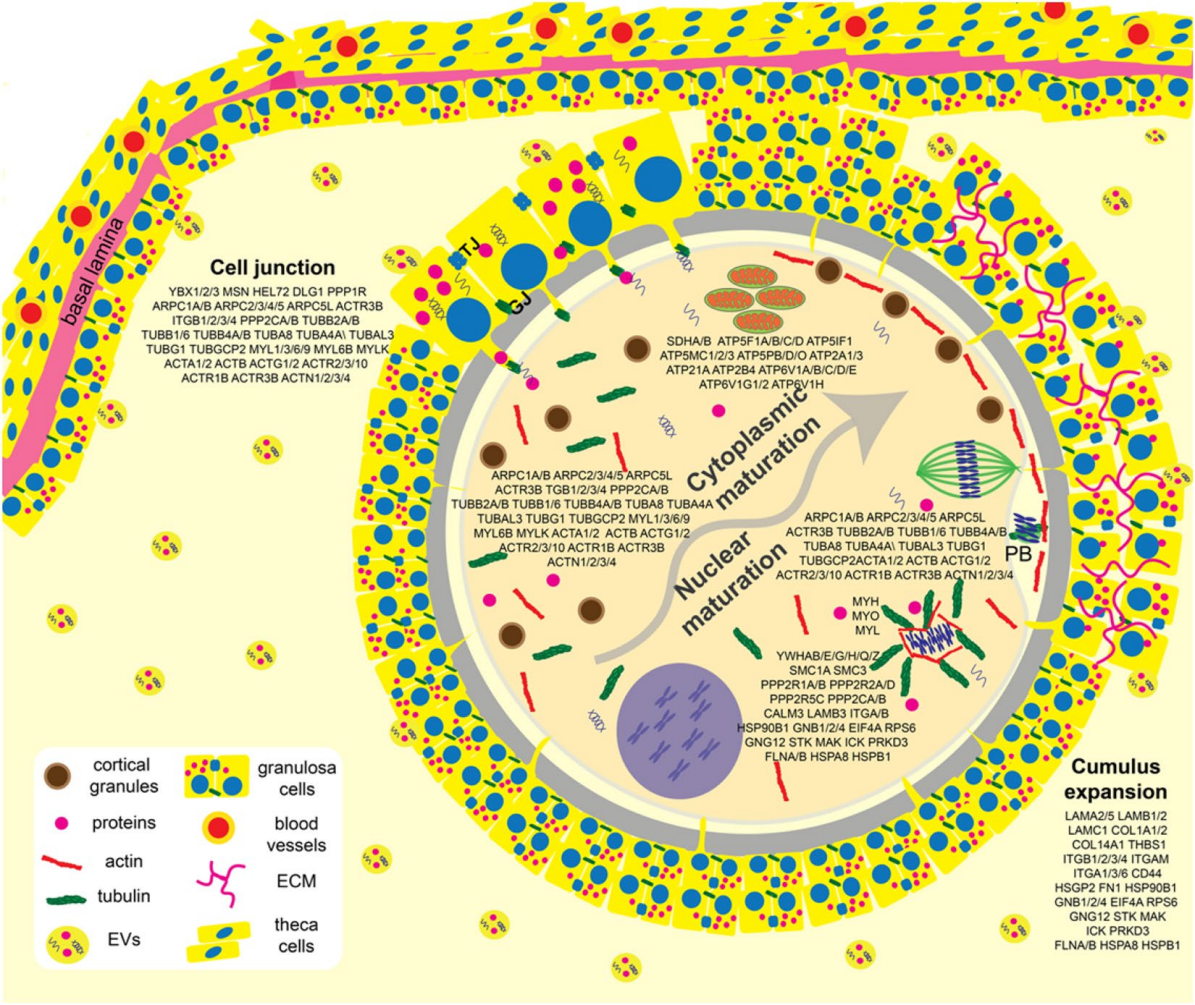
Representative ovarian follicle with COC, summarizing oocyte cytoplasmic and nuclear maturation steps and participating proteins that could be delivered by ffEVs. PB = polar body, TJ = tight junction, GJ = gap junction, ECM = extracellular matrix.

Through these analyses, twenty-four GO protein classes were identified to have roles in modulating response to cryopreservation, including oxidoreductases, cytoskeletal proteins, and chaperones (Fig. 2b). Chaperone proteins, such as the heat shock proteins (HSPs) are known to respond to a series of stresses, including sub- or supra physiological temperatures, toxic substances, extreme concentration of ions and other osmolytes^70,71^, conditions which are encountered by the oocyte during cryopreservation. Nine HSPs were abundantly detected in the cat ffEVs (HSPE1, HSPD1, HSP90AB1, HSPB1, HSP90AA1, HSPA8, LOC105260573, HSPA4L and HSPA2). Another class of chaperons, the chaperonins, that are necessary for folding actin, tubulin and newly synthesized proteins, playing a role on establishing functional cytoskeleton^72^, were also detected in the cat ffEVs (CCT8, CCT5, TCP1, CCT7, CCT2, CCT6B, CCT6A, CCT3 and CCT4). Additionally, many identified proteins from ffEVs are known to regulate oocyte maturation processes, including the Germinal Vesicle Breakdown (GVB), spindle migration and rotation, chromosome segregation, polar body extrusion, cumulus cells expansion, cell junctions, and cytoplasm maturation^63,69,73–76^ (Fig. 3). Taken together, we postulated that the transfer of these ffEV proteins to the oocytes prior to vitrification and/or during thawing could improve immature oocyte cryotolerance.

### Follicular fluid EVs are taken up by the immature COCs and deliver their membrane protein and lipid contents to the COCs

**Figure 4:**
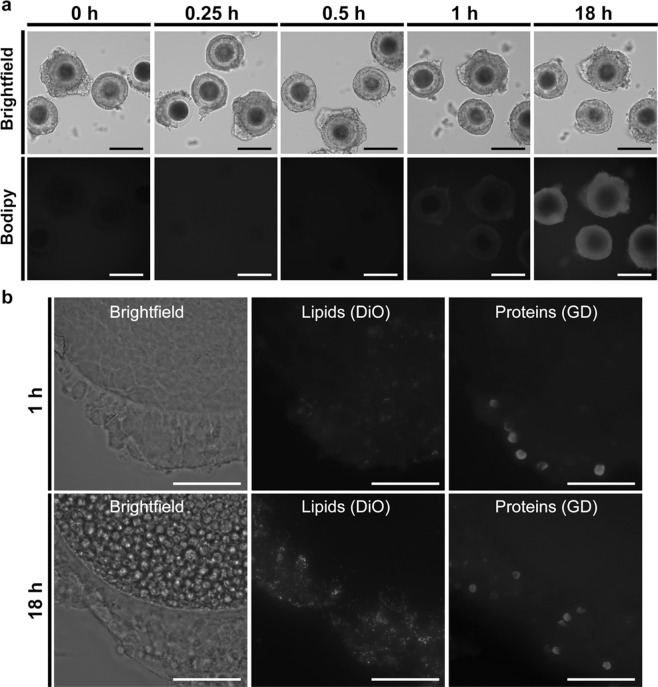
COCs uptake of ffEVs. In **a**, BODIPY labeled ffEVs uptake was evaluated before (0 hr), or following 0.25 h, 0.5 h, 1 h, or 18 h co-incubation with COCs. Fluorescence image in **a** showing uptake of ffEVs by the cumulus cells in a time dependent manner. In **b**, transfer of lipid (DiO) and membrane-bound protein (GD) labeled ffEVs to COCs after 1 or 18 h incubation. Bars = 100 and 25 µm for a and b panels, respectively.

Extracellular vesicle entry and cargo release into cells has been proposed to occur via endocytosis, phagocytosis, micropinocytosis and/or through direct EV-plasma membrane fusion (reviewed in^77^). Lipid dyes, such as the fluorescent neutral lipid BODIPY TR ceramide and the carbocyanine dyes DiO and DiL, and amine groups binding dyes, such as the Ghost dye UV (GD), have already been used to investigate the binding and delivery of EVs lipids and proteins, respectively, to cells^78–80^. To determine whether cat ffEVs were taken up by COCs, COCs were incubated with BODIPY TR Ceramide labeled ffEVs (1.5 ×10^7^ particles/ml) and imaged at 15 minutes, 30 minutes, 1 hour and 18 hours. Uptake of ffEVs by COCs was first detected after 30 minutes incubation and, at 1 hour, all analyzed COCs had the red fluorescence in their cumulus cells layer (Fig. 4a) which was not observed in the no ffEVs controls (Supplementary Fig. 5). Next, COCs were incubated with DiO and GD labeled ffEVs (1.5 ×10^7^ particles/ml) and analyzed at 1 hour and 18 hours for lipid and membrane-bound protein uptake, respectively. Similar to the BODIPY staining, both lipids and membrane-bound proteins were detected in the cumulus cells at 1 hour and maximized at 18 hours (Fig. 4b). However, labeled lipids and membrane-bound proteins were not detected in the oocytes even after an 18 hour incubation period. This finding is consistent with a bovine study in which it was reported that ffEVs were taken up by cumulus cells but were not observable via confocal microscopy in the oocyte after 16 hours incubation^33^. In another study, oviductal EVs were observed to be taken up by domestic dog oocytes after 72 hours incubation^81^. In the cow study as in the present, it was not conclusively determined if the lack of observable labeled proteins/lipids in the oocyte and/or its plasma membrane was due to their absence, or if the amount delivered was below the threshold of detection by confocal and widefield fluorescence microscopy, respectively. Future studies using higher resolution microscopy, such as the stochastic optical reconstruction microscopy (STORM) could help answer this. Nevertheless, based on these results we selected the 1 h time for pre-incubation of COCs with ffEVs for the vitrification study.

### Incubation with ffEVs improves oocyte meiosis resumption after vitrification

**Figure 5:**
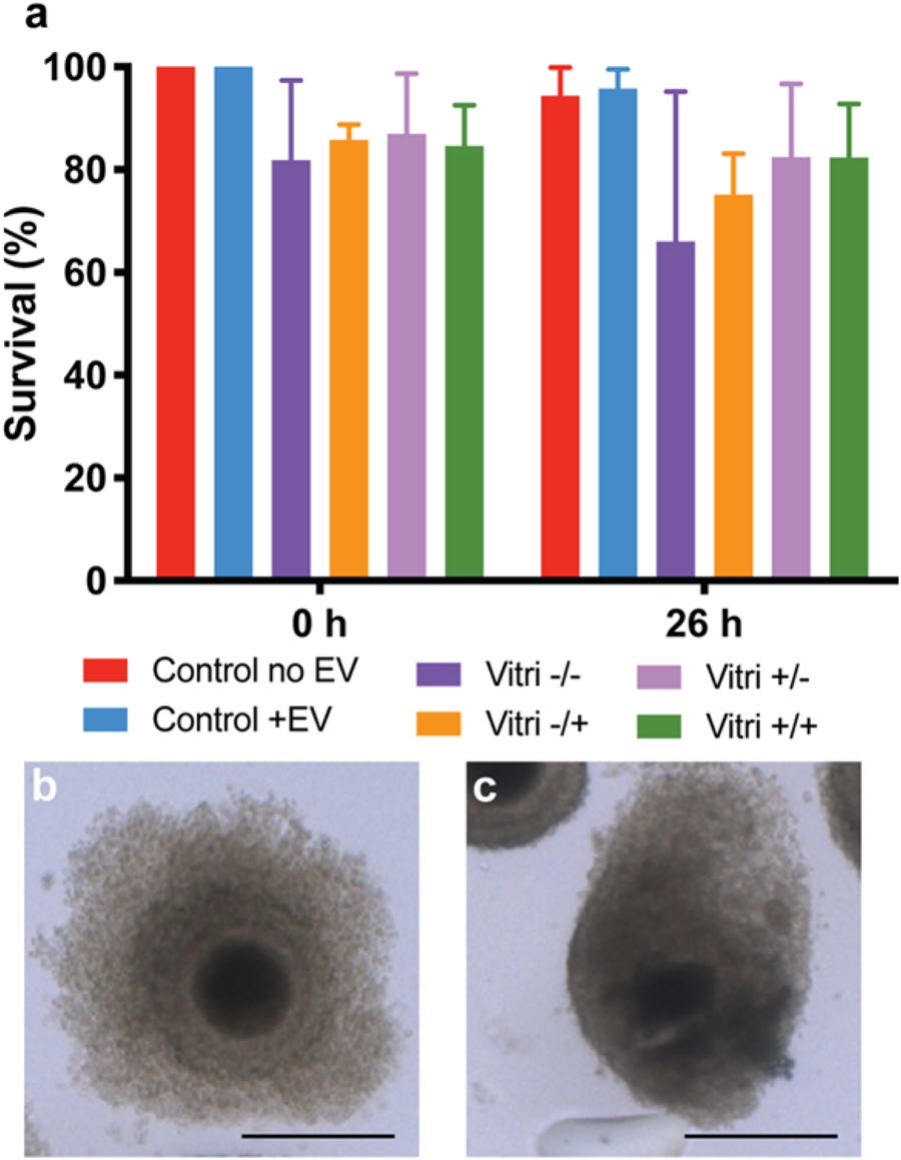
In **a**, survival rate of non-vitrified (Control) and vitrified oocytes in the presence or absence of ffEVs. Data is present as mean ± SD. In **b** a normal surviving oocyte and in **c** a non-surviving oocyte. Bars = 100 µm.

**Figure 6:**
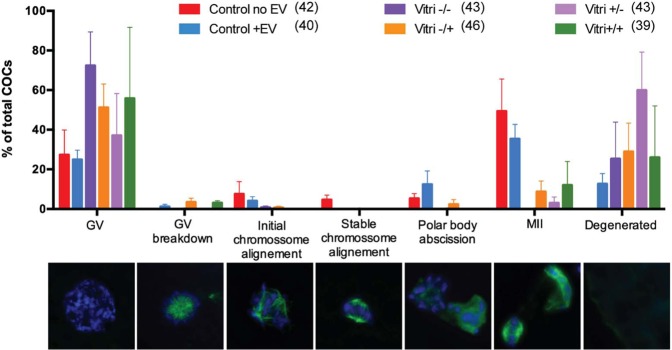
Oocyte maturation stages of non-vitrified (Control) and vitrified COCs (Vitri), in the presence (+EV) or absence (-EV) of ffEVs, detected at 26 h. Bellow each stage in the graph are the corresponding representative images of the progression of oocyte meiotic stages with chromatin content (Hoechst 33342, blue) and tubulin (green). From left to right are germinal vesicle (GV), germinal vesicle breakdown (GV breakdown), initial chromosome alignment, stable chromosome alignment, polar body abscission, metaphase II (MII) and degeneration. No significant differences were noted between controls and vitrified oocytes (Friedman non-parametric analysis of variance, p > 0.05), regardless of the presence of ffEVs previously to or during thawing.

We next sought to evaluate the impact of ffEV supplementation to cat oocyte survival immediately and 26 h post-vitrification. There was no significant difference in oocyte viability among groups (Fig. 5). When comparing individual oocyte maturation stages, there also were no significant difference among treatment groups (Fig. 6).

**Figure 7:**
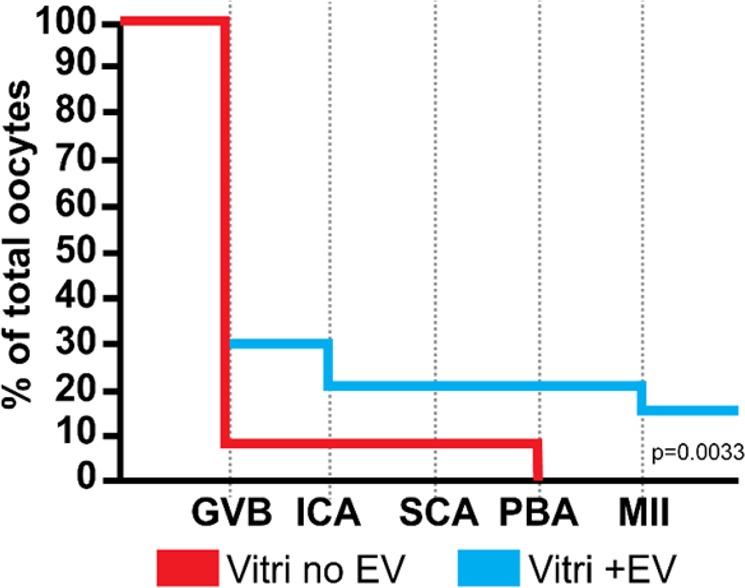
Activation of meiotic resumption plot of vitrified oocytes in the absence (Vitri no EV) or presence of ffEVs (Vitri + EV) through the experimental time point. Note the higher initiation of oocyte maturation in the Vitri +EV group (ChiSquare, p = 0.0033). GVB, germinal vesicle breakdown; ICA, Innitial chromosome alignment; SCA, Stable chromosome alignment; PBA, Polar body abscission; MII, Metaphase II.

Vitrification compromised the ability of the oocytes to undergo meiotic resumption (GVB to MII) (21 ± 14.1 *vs* 74.5 ± 4.9%, ChiSquare, p < 0.0001, average ± SD of all vitrified treatments and the two fresh controls, respectively). However, the presence of ffEVs before and/or after vitrification improved meiotic resumption in vitrified COCs (28.3 ± 13.1 *vs* 8.6%, ChiSquare, p = 0.0033, Fig. 7). Furthermore, vitrified oocytes only reached metaphase II (MII) when ffEVs were present (17.9%), although these values were still much lower than the fresh control oocytes (42.5%). Reduced maturation due to vitrification damage was also described for vitrified immature COCs in several species, including the cat, bovine, ovine, horse and human^3,11,20,82,83^.

The improved meiotic resumption of vitrified oocytes co-incubated with ffEVs could be explained by the delivery of different proteins, RNAs, and lipids from the ffEVs to the COCs. Potential modulating proteins can be broadly grouped into four categories based on their function: cell-cell communications, meiosis resumption, structural stabilization, and metabolism. The bidirectional communication between the oocyte and the granulosa cells via gap junction permits follicular and oocyte development and is mediated by a network of cellular junctions^76^. In the present study, we demonstrated that proteins regulating the formation of tight junctions (such as PP2A, DLG3, ZONAB, ERM, SYNPO, different actin isoforms, Arp2/3, Integrin, myosin II and TUBA) are also present in the cat ffEVs. Because proteins from tight junctions can also regulate gap junction formation, it is possible that these proteins^84,85^ could play roles in maintaining the communication between the granulosa cells and, possibly, between granulosa cells and the oocytes, after cryopreservation.

Follicular fluid EV proteins with potential roles in modulating meiosis resumption include those involved in cumulus cell expansion, nuclear envelope breakdown, and spindle stabilization. Previous studies have shown that ffEVs are taken up by bovine COCs and enhance cumulus cell expansion^33^. This effect is possibly mediated by proteins involved in MAPK and PI3K pathways^76^, many of which were found in cat ffEVs. Cumulus cell expansion is also dependent on cumulus extracellular matrix formation and composition^69^. Here, we identified ECM components and receptors in the cat ffEVs (Fig. 2a), previously demonstrated to be required for COCs matrix formation in bovine granulosa cells^76^. It is also known that MAPK and PI3K pathways are important for the meiotic resumption in the oocyte^86^. In the present study, several proteins involved in the MAPK and PI3K signaling pathways, including SMC1A, SMC3, PP2Rs, CALM3, HSP90B1, HSPA8, HSPB1, EIF4A, MAK, and ICK, were present in cat ffEVs. Likewise, miRNAs regulating the same pathways have been found in bovine, equine and human ffEVs^32,33,37,38,87,88^. Therefore, these proteins and miRNAs could play important roles on oocyte meiotic resumption. For example, the Arp2/3 complex is essential for F-actin shell nucleation and consequent nuclear membrane fragmentation, leading to the nuclear envelope breakdown (NEB) in starfish^65^. Components of the Arp2/3 complex that play roles in actin cytoskeleton formation were abundant in the cat ffEVs and could also contribute to the higher meiosis resumption of vitrified COCs in the presence of ffEVs.

Improved mitochondrial function could also contribute to the higher meiotic resumption of ffEV supplemented vitrified oocytes. When porcine oocytes were incubated with the mitochondrial activity inhibitor Carbonyl cyanide p-trifluoromethoxyphenylhydrazone (FCCP), a significant reduction in the membrane potential and first polar body extrusion was observed^89^. Similarly, FCCP reduced the percentage of oocyte with nuclear maturation, normal spindle formation and chromosome alignment in mice^90^ demonstrating the importance of mitochondrial activity for normal oocyte maturation. The cat ffEVs had, at least, 26 proteins that are part of the oxidative phosphorylation and could modulate mitochondria function, improving vitrified oocyte maturation. It is likely that ffEVs also contributed to cell structural recovery and to stress response following vitrification. Vitrification was previously described to increase the number of abnormal spindle in MII oocytes from sow, cow, woman, mouse, horse and cat^18,54,91–93^. In our study, no abnormal MII spindles were observed in the non-vitrified controls or in the vitrified oocytes that were incubated with ffEVs. It may be that ffEVs delivered factors such as F-Actin, Arp2/3, PIR121, VCL, actin, myosin, ERM, PI4P5K, CFN, FN1, UTG, GSN, MLC, among others, that could positively affect the spindle stabilization of vitrified oocytes after vitrification. Likewise, an increased stress induced response through HSPs and chaperonins could also prevent spindle abnormalities. In the mouse, the oocyte has maximized heat shock response during the growth period, which declines when they acquire their full size and is shut off in later stages of follicular maturation, around the GVB, which could explain why mammalian oocytes are sensitive to thermic stress^94,95^. Reduced polar body extrusion and increased abnormal spindle assemblies have been observed when the modulation of the chaperonin TCP1 function was depleted in mouse oocytes (by siRNA silencing of its modulator *Txndc9*)^72^.

## Conclusion

In summary, the present study is the first to characterize protein content of cat ffEVs. Our results showed that ffEVs are enriched in proteins that can play roles in regulating follicle growth, oocyte energy metabolism, oocyte maturation, stress response and cell-cell communication, suggesting that ffEVs may play important roles in the crosstalk that occurs between the somatic and germline follicular components. We also demonstrated, for the first time, that ffEVs improve meiotic resumption of vitrified COCs and can be used as a tool to improve gamete cryopreservation. The next steps are to identify mechanisms by which ffEVs regulate follicle and oocyte development. Furthermore, it is well established that the plasma membrane permeability modulates several types of cell injury associated with cryopreservation, including volume changes due to osmotic stress and CPA toxicity^96,97^. Therefore, investigations on the role of ffEVs lipids on COC function and cryopreservation are also required.

## Materials and methods

### Reagents

All reagents were purchased from Sigma Aldrich (St. Louis), unless otherwise stated.

### Follicular fluid EV (ffEV) isolation and oocyte collection

Domestic cat ovaries voided of corpora hemorrhagica and/or lutea (4 months to 3 years old) were opportunistically collected from local veterinary clinics after routine ovariohysterectomy and transported at 4 °C to the laboratory within 6 hours of excision. No additional permissions were required since these biological materials were designated for disposal via incineration. After being washed three times in Phosphate Buffer Saline solution (PBS, GIBCO, USA), follicular fluid was aspirated from antral-stage follicles (1–8 mm diameter), centrifuged at 2,000 × g at room temperature for 30 minutes to remove cells and debris. The supernatant was then mixed with 500 μL of the Total Exosome Isolation Reagent (Invitrogen, USA) and incubated overnight at 4 °C. The samples were centrifuged at 10,000 × g for 1 hour, and the pellet resuspended in 50 μL of PBS. ffEVs were then aliquoted, and stored at −20 °C until use. Each ffEV aliquot was only thawed once immediately prior to utilization, to avoid multiple freeze/thaw cycles.

Oocytes were collected from spayed domestic cat ovaries (>10 months old). Ovaries were washed in handling medium composed of MEM eagle with 100 U ml^−1^ penicillin G, 10 mg ml^−1^ streptomycin sulfate, 100 mM pyruvate, 25 mM HEPES, and 4 mg ml^−1^ bovine serum albumin. Oocytes were collected via dicing of the ovarian cortex with a scalpel blade. Homogenously dark, circular oocytes with at least two layers of surrounding cumulus cells were selected for use.

### Follicular fluid EV quantitation

Nanoparticle tracking analysis was done using the ZetaView S/N 17–332 (Particle Metrix, Meerbusch, Germany) and data analyzed using its software (ZetaView 8.04.02) by Alpha Nano Tech (Research Triangle Drive, NC, USA) as previously described^98^. For each replicate (n  =  4, pooled frozen-thawed samples from 1–4 individuals, totaling 9 cats), ffEVs sample (1 ml) was diluted 100X in PBS, loaded into the cell, and the instrument measured each sample at 11 different positions throughout the cell, with three cycles of readings at each position. The pre-acquisition parameters were: sensitivity of 85, frame rate of 30 frames per second (fps), shutter speed and laser pulse duration of 100, temperature of 19.81 °C, and pH of 7.0. Post-acquisition parameters were set to a minimum brightness of 22, a maximum area of 1000 pixels, and a minimum area of 10 pixels^98^. All parameters (temperature, conductivity, electrical field, and drift measurements) were documented for quality control. After software analysis, the mean, median, and mode (indicated as diameter) sizes, as well as the concentration of the sample, were calculated, excluding outliers^98^. The number of particles per particle size curves was created using quadratic interpolation^98^.

### Proteomic analyses

Follicular fluid EVs (n  =   7 cats) were pooled, frozen at −20 °C. Proteins were extracted and prepared via single-pot, solid phase-enhanced sample-preparation (SP3) technology^99^ by Bioproximity LLC (Chantilly, VA), and analyzed using ultraperformance liquid chromatography and tandem mass spectrometry (UPLC - Thermo Easy-nLC 1200 fitted with a heated, 25 cm Easy-Spray column – MS/MS - Thermo Q-Exactive HF-X quadrupole-Orbitrap mass spectrometer). The peptide dataset (mzML format) were exported to Mascot generic format (mgf) and searched using X!!Tandem^100^ using both the native and k-score scoring algorithms^101^, and by OMSSA^102^. RAW data files were compared with the protein sequence libraries available for the domestic cat (*Felis catus*, taxa 9685). Label free quantification (MS1-based) was used and peptide peak areas were calculated using OpenMS^103^. Proteins were required to have one or more unique peptides across the analyzed samples with *E*-value scores of 0.01 or less.

### Functional GO clustering

Data Entrez Gene IDs were mapped for all identified proteins using the R package rentrez (ver 1.2.1)^104^. The background dataset for all analyses was the cat (*Felis catus*) genome. Gene ontology (GO) analyses were performed using KEGG mapper^59,60,62^ (https://www.genome.jp/kegg/mapper.html) web-based software and the use of figures in the present manuscript were granted by Kanehisa Laboratories under reference 200029. For enrichment analysis, the cut off was set to *p*  <  0.05. The Cytoscape 3.5.1 plugin ClueGO^58^ was used to visualize interactions of EVs proteins and networks integration, by GO terms “biological processes”, “molecular function” and “cellular components” using the *Felis catus* genome. The evidence was set to “Inferred by Curator (IC)”, and the statistical test was set to a right-sided hypergeometrical test with a κ score of 0.7–0.9 using Bonferroni (step down). The function “GO Term fusion” was selected, the GO term restriction levels were set to 3, and a minimum of two genes or 5% genes in each GO term was used.

### Follicular Fluid EV transmission electron microscopy

TEM preparation and imaging was performed at the Alpha Nano Tech (Research Triangle Drive, NC, USA) on isolated EVs, stored at -20 °C until analysis. Briefly, Formvar coated carbon coated TEM grids (Electron Microscopy Sciences) were floated on 10 µL of sample drop for 10 minutes, washed two times with water by floating on the drop of water for 30 seconds, and negatively stained with uranyl acetate (2%) by floating on the drop of stain for 30 seconds. The grids were blot dried with Whatman paper and imaged on a Jeol JEM1230 transmission electron microscope (TEM).

### Follicular Fluid EV Uptake Testing

Follicular fluid EVs (n = 3 cats) were stained by phospholipid bilayer BODIPY^®^ TR Ceramide (Invitrogen, USA) or by a combination of DiO (Thermo-Fisher) and Ghost dye UV (GD, Tonbo Biosciences). Briefly, 5 µM BODIPY TR Ceramide stock solution in DMSO or 1 µg mL^−1^ of DiO + 1 µL mL^−1^ of GD, were incubated with 50 μl of total EVs for 30 minutes at room temperature (BODIPY) or at 37 °C (DiO + GD). Unbound dye was removed by Exosome Spin Columns (Invitrogen, USA), following manufacturer’s instructions. For negative controls, the dye samples without EVs were passed through the Exosome spin columns, and then used in uptake experiments.

Freshly collected domestic cat cumulus-oocyte complexes (COCs, n = 3 animals, 100 oocytes) were co-incubated in 50 µl droplets of handling medium, immersed in mineral oil, in the absence or presence of 1.5 ×10^7^ particles ml^−1^ dyed ffEVs. Oocytes (5–7/droplet) were imaged at 10, 20, 40 and 100 × under a fluorescence microscope (EVOS FL auto 2, Invitrogen, USA) after 0, 15, 30, 60, and 1080 min (18 hr) co-incubation at 5% CO_2_ and 38 °C.

### Oocyte Cryopreservation and Thawing

COCs were cryopreserved using a protocol modified from Comizzoli *et al*. (2009)^105^ and Colombo *et al*. (2019)^16^. Briefly, COCs were transferred in small groups (7–11 oocytes) via a mouth pipette to an equilibration solution, consisting of handling medium supplemented with 7.5% (v/v) ethylene glycol and 7.5% (v/v) dimethylsulfoxide for 3 minutes on ice. COCs were then briefly exposed to vitrification solution containing 15% ethylene glycol, 15% DMSO, and 0.5 M sucrose in handling medium for 30 seconds before being loaded in minimal volume on a Cryotop (Kitazato, Japan) and plunged into liquid nitrogen according to manufacturer’s instructions. COCs were thawed by passing them through a sucrose gradient (1.0, 0.5, 0.25, and 0 M) of warmed (38.5 °C) handling medium with or without 1.5 ×10^7^ particles ml^−1^ ffEV.

### *In Vitro* Maturation

*In vitro* maturation of cat oocytes was performed using a method previously described for the domestic cat^106^. Briefly, 50 µl droplets of Quinn’s Advantage Protein Plus Blastocyst Medium, supplemented with 1 µg ml^−1^ follicle stimulating hormone (Folltropin, Vetoquinol, USA), and luteinizing hormone (Lutropin V, Vetoquinol, USA), were equilibrated under mineral oil at 5% CO_2_ and 38 °C for at least 2 hours before use. Fresh or frozen-thawed cat COCs were transferred in groups of 14–28 oocytes to each droplet and incubated for 24 hours. Following IVM, oocytes were washed in PBS containing EDTA (0.1 mM), EGTA (0.1 mM), Imidazole (50 mM), 4% Triton-X, PVP (3 mg mL^−1^) and PMSF (24 µM). During this step, oocytes were rapidly pipetted up and down to remove cumulus cells, then fixed overnight in 4% paraformaldehyde in PBS at 4 °C.

Oocyte survival, defined as maintaining normal morphology ﻿with spherical shape, intact membranes, clear zone without rupture, and uniform cytoplasm, was evaluated immediately after thawing (0 h) and following *in vitro* maturation (26 h) via light microscopy^107^.

### Oocyte Spindle Staining and Imaging

After fixation, oocytes were washed in washing buffer consisting PBS with 2.5% (v/v) normal goat serum and 0.5% triton X, then blocked for 30 min at 38 °C in PBS with 5% normal goat serum and 0.5% Triton X before incubation with rabbit anti-alpha tubulin (ab18251, Abcam) at 1:125 dilution in washing buffer for 1 hour at 38 °C. After washing, oocytes were incubated for 1 hour at 38 °C in goat-anti-rabbit IgG (1:200 dilution) and Hoechst 33342 (5 µg mL^−1^, Invitrogen) washed and placed into a small drop of ProLong Glass Anti-fade mounting solution (Invitrogen P36980) on a glass slide, and imaging under a fluorescent microscope (EVOS FL auto 2). Oocytes were imaged under 1,000 × magnification using a fluorescence microscope (EVOS FL auto 2, Invitrogen, USA) to observe microtubule organization, and staged as previously described with some modifications^108^.

### Experimental Design

#### Study 1: Characterization of cat follicular fluid

Follicular fluid was collected and pooled from 1–4 individuals in domestic cat (n  =  9). The amount of EVs in pooled follicular fluid was then quantified by Nano tracking analysis and its presence confirmed by transmission electron microscopy. Pooled ffEV samples from 7 of same 9 cats were subjected to proteomics analysis and uptake testing.

#### Study 2: Effect of follicular fluid EV on oocyte vitrification

**Figure 8:**
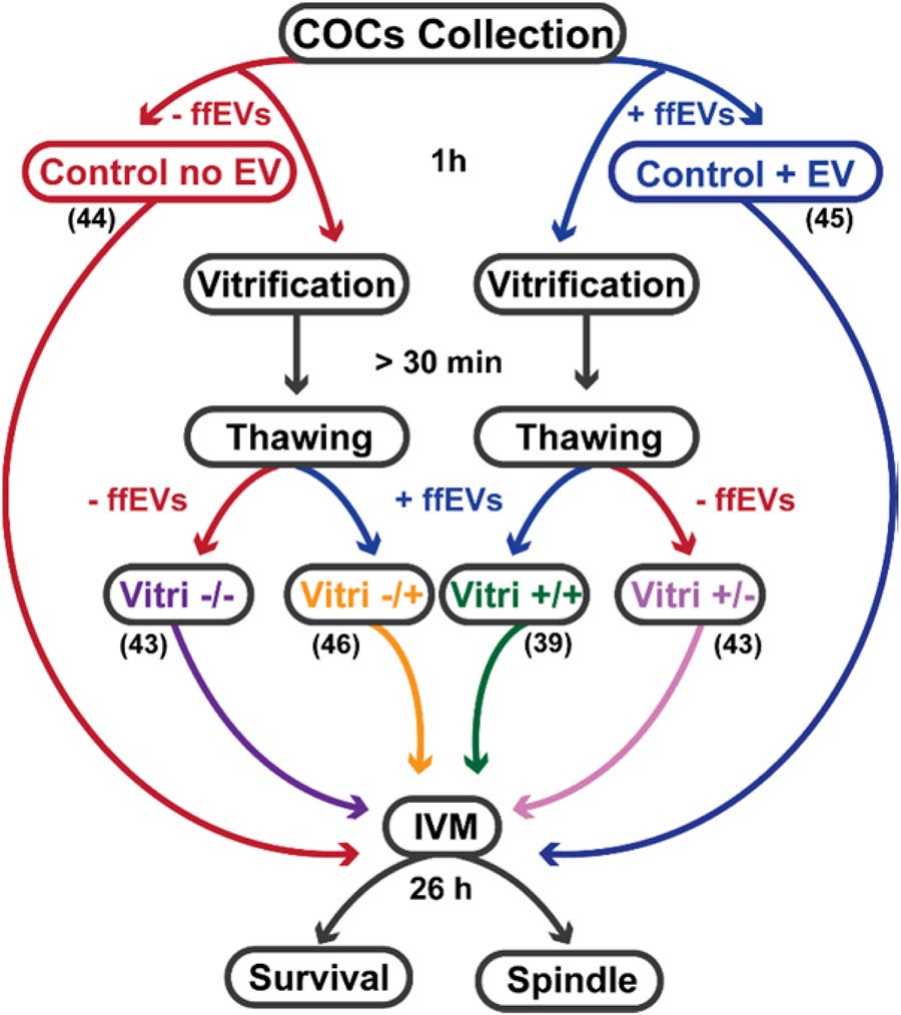
Experimental design of ffEVs-COCs incubation, vitrification and thawing. Number written in each group indicates the number of oocytes used for vitrification/maturation experiments.

Domestic cat COCs (n = 15 cats, 267 oocytes) were co-incubated in the absence (with PBS) or presence of 1.5 ×10^7^ particles ml^−1^ unlabeled ffEV (diluted in PBS) at 5% CO_2_ and 38 °C for 1 hour. A subset of oocytes was then *in vitro* matured as fresh controls (n = 44 oocytes without ffEV, n = 45 with ffEV), and the remaining oocytes were vitrified in the presence or absence of ffEV at the same concentration in equilibration and vitrification solutions. COCs were maintained in liquid nitrogen for at least 30 minutes before being thawed (with or without ffEV) and subjected to *in vitro* maturation (Fig. 8).

### Statistical Analysis

Data are presented as means ± standard deviation (SD). Comparisons in oocyte viability and maturation stage among treatments were evaluated by the Friedman non-parametric analysis of variance (GraphPad PRISM 7, GraphPad Software, USA). Comparisons in oocyte maturation between control and vitrification group and between vitrification with/without ffEV treatment were evaluated via Kaplan-Meier (displayed as log-rank prob > ChiSquare) with oocyte maturation stage coded as a continuous variable (germinal vesicle = 0, germinal vesicle breakdown = 1… Metaphase II = 6) in JMP Pro 12 (SAS Institute Inc., USA). Differences were considered significant at p < 0.05.

## Supplementary information

Supplementary files.

Supplementary data 1.

## Data Availability

The authors declare that all data supporting the findings of this study are available within the article, Supplementary Files, or from the corresponding author upon reasonable request. UPLC-MS/MS (mzML) file has been deposited in FIGSHARE database under DOI number: 10.6084/m9.figshare.7837331.
