# Supplementary material for: Follicular extracellular vesicles enhance meiotic resumption of domestic cat vitrified oocytes
Source: Sci Rep. 2020 May 25;10:8619. doi: 10.1038/s41598-020-65497-w (PMC7248092; doi:10.1038/s41598-020-65497-w)
Supplement: Supplementary file 1 — Supplementary files. [file 41598_2020_65497_MOESM1_ESM.docx]

**Follicular extracellular vesicles enhance meiotic resumption of domestic cat vitrified oocytes**

Marcia de Almeida Monteiro Melo Ferraz^a^*^#^, Mayako Fujihara^b#^, Jennifer Beth Nagashima^a^, Michael James Noonan^a^, Miho Inoue-Murayama^b,c^, Nucharin Songsasen^a^

^a^ Smithsonian National Zoo and Conservation Biology Institute, 1500 Remount Road, Front Royal, Virginia 22630, USA.

^b^ Wildlife Research Center, Kyoto University, 2-24 Tanaka-Sekiden-cho, Sakyo, Kyoto 606-8203, Japan

^c^ Wildlife Genome Collaborative Research Group, National Institute for Environmental Studies, 16-2 Onogawa, Tsukuba, Ibaraki 305-8506, Japan

## * Corresponding author

[ferrazm@si.edu](mailto:ferrazm@si.edu)

+1 (540) 635-0494

^#^ The authors contributed equally to the study.

**Supplementary Files**

**Supplementary Figures**


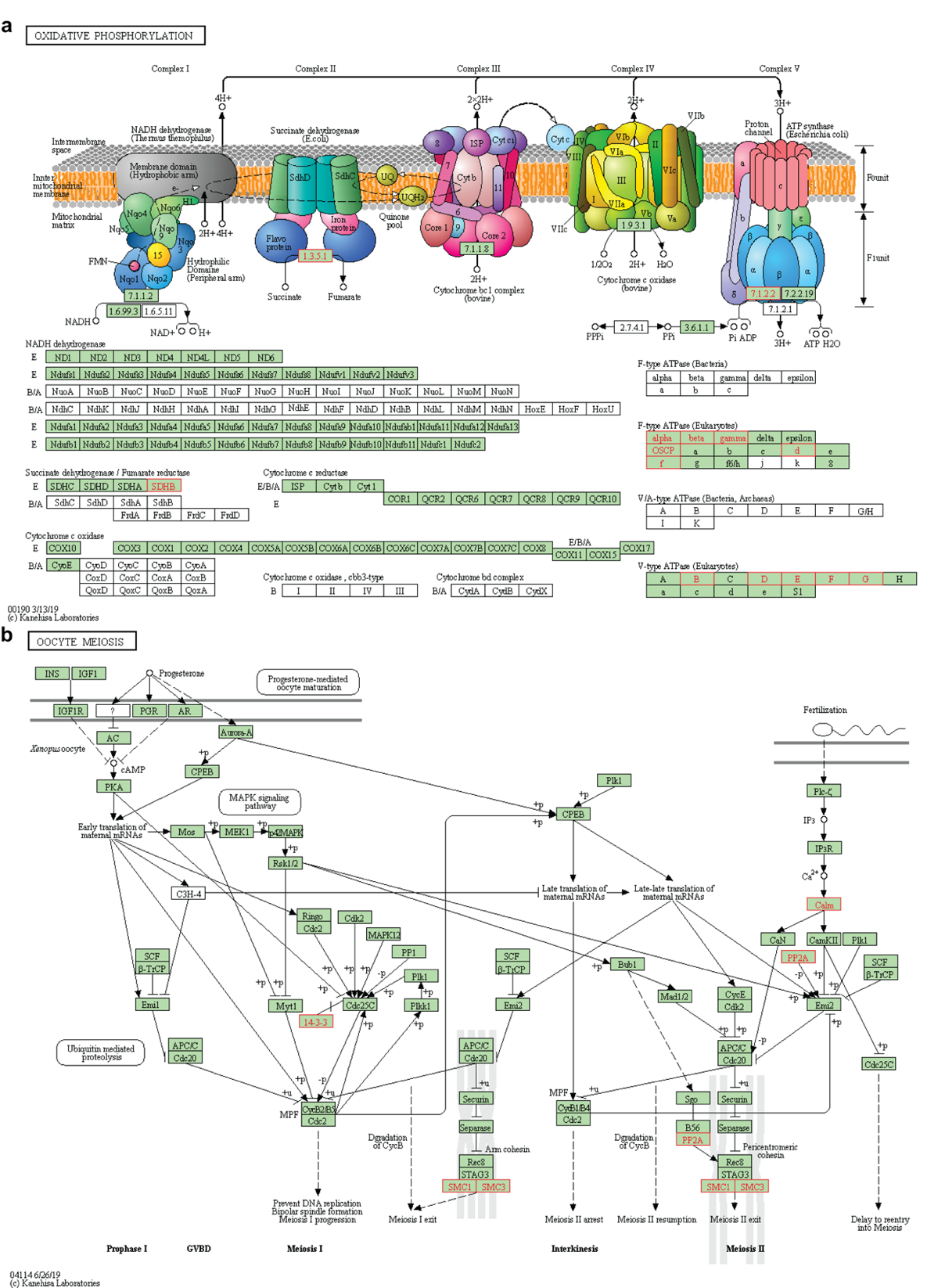


**Supplementary Fig. 1.** KEGG molecular pathways of ffEVs identified proteins performed by KEGG mapper (http://www.genome.jp/ kegg/mapper)^1^. In **a** KEGG pathways related to oxidative phosphorylation and in **b** oocyte meiosis. Note that proteins present in ffEVs are shown in red.

**
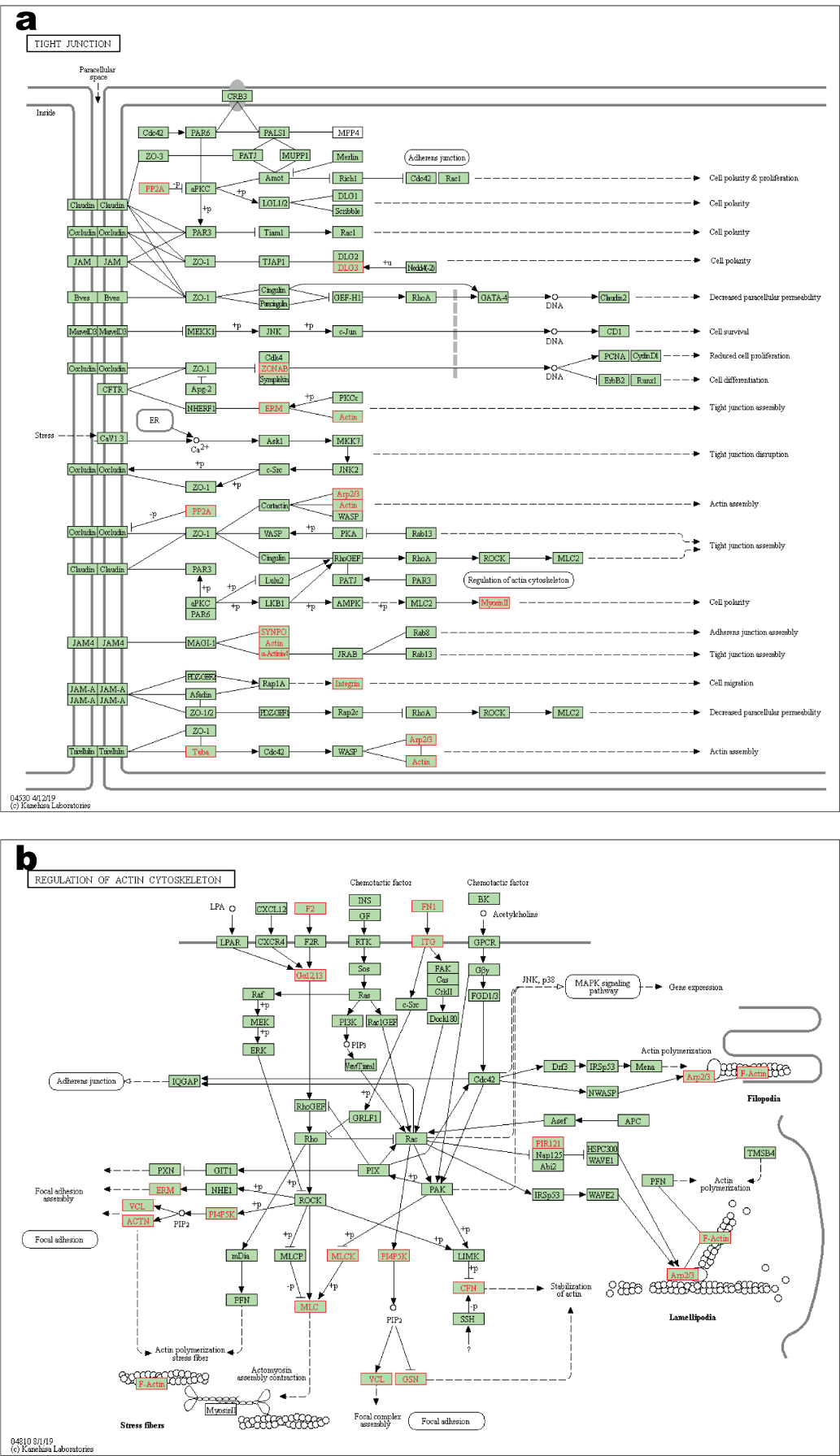
**

**Supplementary Fig. 2.** KEGG molecular pathways of ffEVs identified proteins performed by KEGG mapper (http://www.genome.jp/ kegg/mapper)^1^. In **a**, KEGG pathways related to tight junction and in **b** to regulation of actin cytoskeleton. Note that proteins present in ffEVs are shown in red.


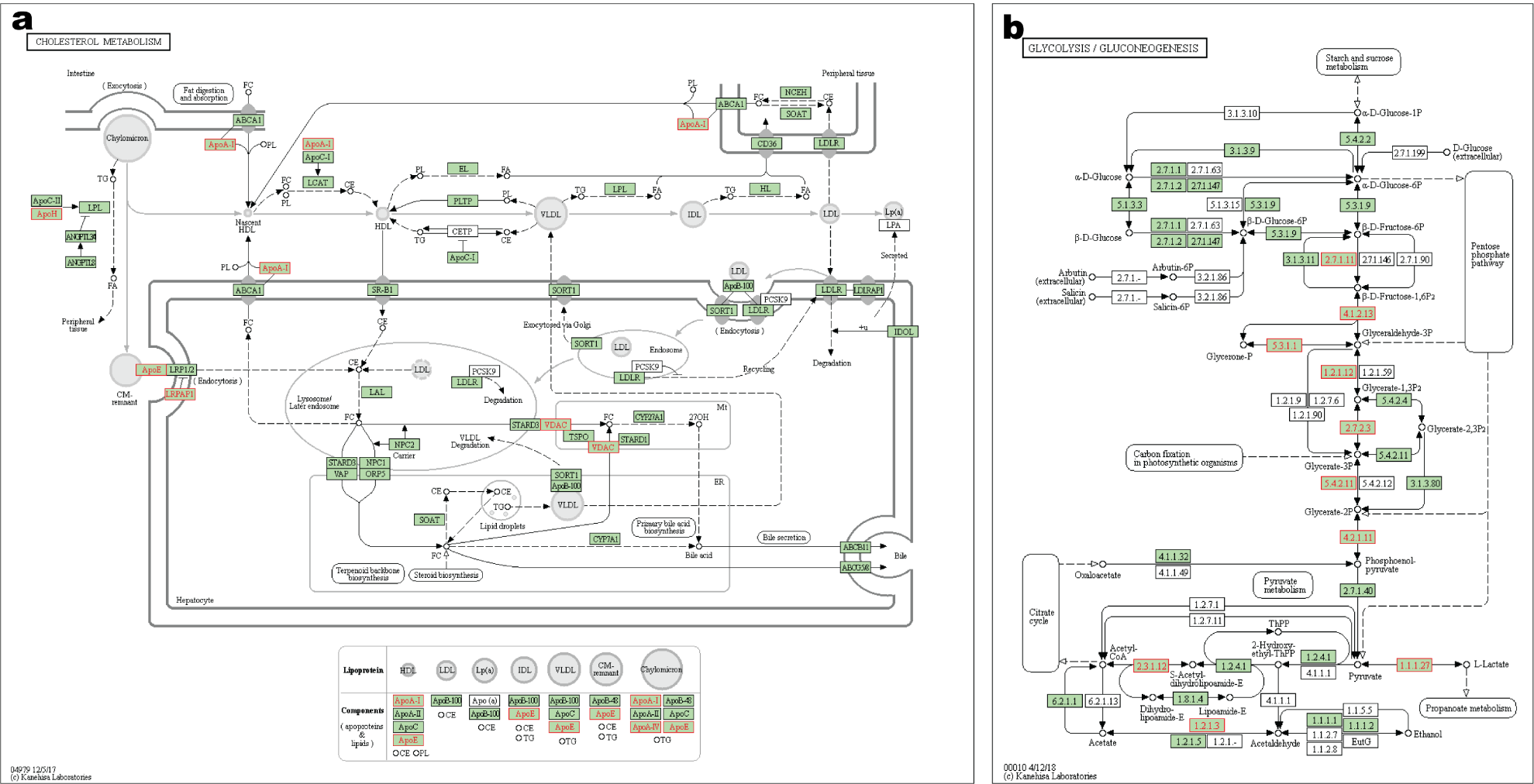


**Supplementary Fig. 3.** KEGG molecular pathways of ffEVs identified proteins performed by KEGG mapper (http://www.genome.jp/ kegg/mapper)^1^. In **a**, KEGG pathways related to cholesterol metabolism and in **b** to glycolysis/gluconeogenesis. Note that proteins present in ffEVs are shown in red.


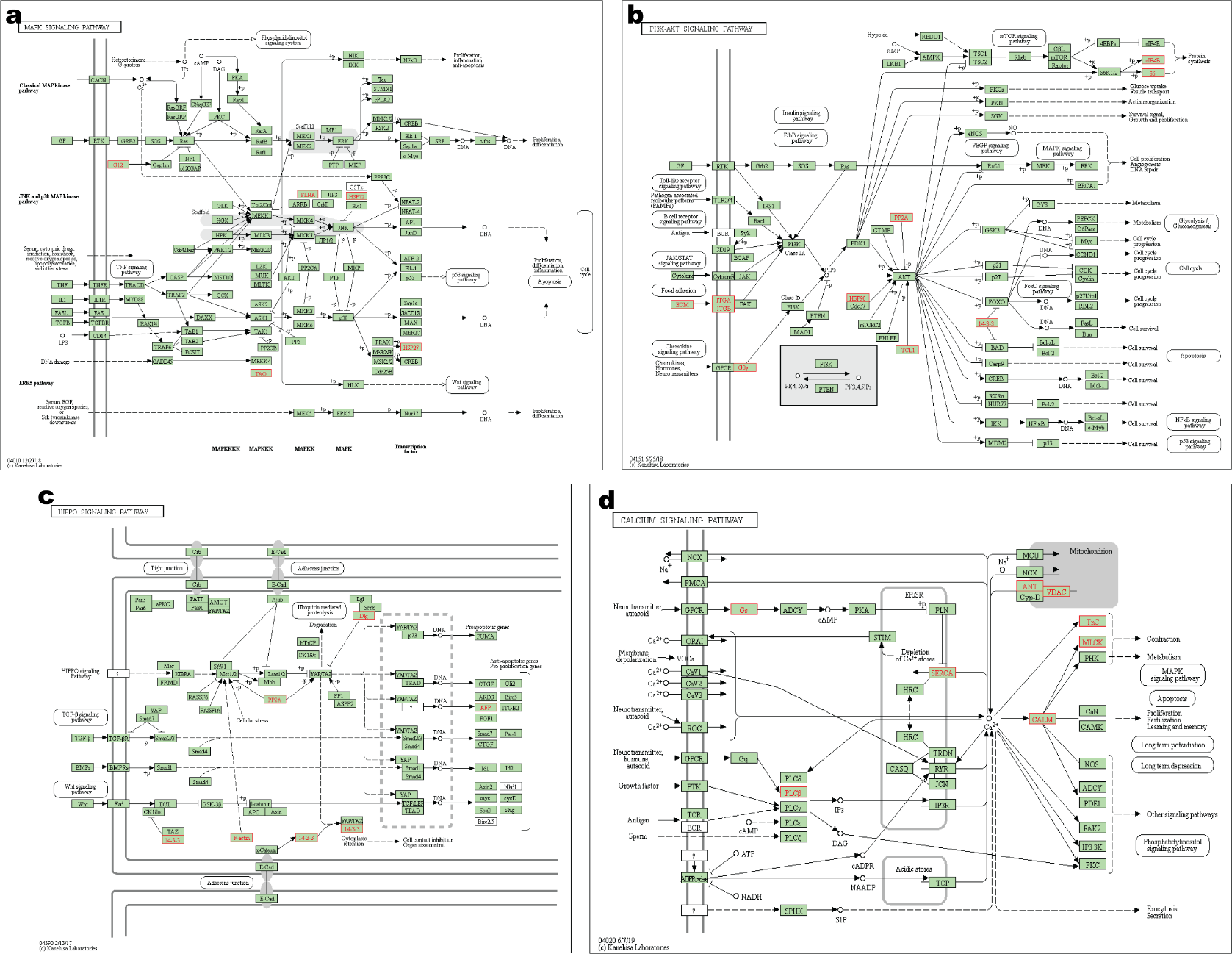


**Supplementary Fig. 4.** KEGG molecular pathways of ffEVs identified proteins performed by KEGG mapper (http://www.genome.jp/ kegg/mapper)^1^. In **a**, KEGG pathways related MAPK signaling pathway, in **b** to PI3K-AKT signaling pathway, in **c** HIPPO signaling pathway and in d Calcium signaling pathway. Note that proteins present in ffEVs are shown in red.


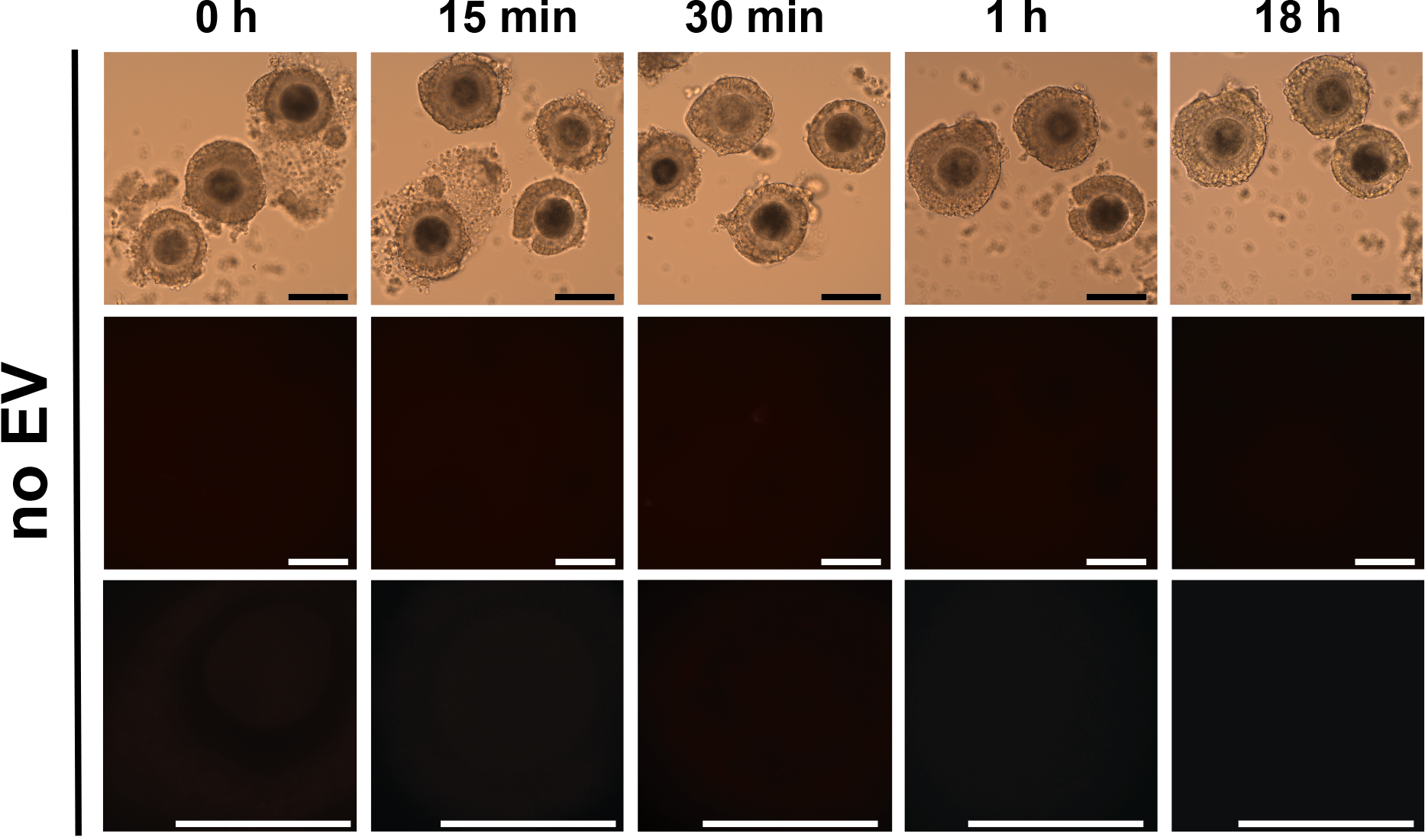


**Supplementary Fig. 5.** Negative control for COCs uptake of ffEVs and cargo transfer, showing no red fluorescence of oocytes incubated in the absence of labeled ffEVs. Middle panel are fluorescence images at 40x magnification and bottom panel at 200x magnification. Scale bars = 100 µm.

**Reference**

1. Kanehisa, M. & Sato, Y. KEGG Mapper for inferring cellular functions from protein sequences. *Protein Sci.* 1–18 (2019). doi:10.1002/pro.3711
